# A deep graph convolutional neural network architecture for graph classification

**DOI:** 10.1371/journal.pone.0279604

**Published:** 2023-03-10

**Authors:** Yuchen Zhou, Hongtao Huo, Zhiwen Hou, Fanliang Bu

**Affiliations:** School of Information Network Security, People’s Public Security University of China, Beijing, China; Chunghwa Telecom Co. Ltd., TAIWAN

## Abstract

Graph Convolutional Networks (GCNs) are powerful deep learning methods for non-Euclidean structure data and achieve impressive performance in many fields. But most of the state-of-the-art GCN models are shallow structures with depths of no more than 3 to 4 layers, which greatly limits the ability of GCN models to extract high-level features of nodes. There are two main reasons for this: 1) Overlaying too many graph convolution layers will lead to the problem of over-smoothing. 2) Graph convolution is a kind of localized filter, which is easily affected by local properties. To solve the above problems, we first propose a novel general framework for graph neural networks called Non-local Message Passing (NLMP). Under this framework, very deep graph convolutional networks can be flexibly designed, and the over-smoothing phenomenon can be suppressed very effectively. Second, we propose a new spatial graph convolution layer to extract node multiscale high-level node features. Finally, we design an end-to-end Deep Graph Convolutional Neural Network II (DGCNNII) model for graph classification task, which is up to 32 layers deep. And the effectiveness of our proposed method is demonstrated by quantifying the graph smoothness of each layer and ablation studies. Experiments on benchmark graph classification datasets show that DGCNNII outperforms a large number of shallow graph neural network baseline methods.

## 1 Introduction

In recent years, the rapid development of Convolution Neural Networks (CNNs) [1] have achieved impressive results in many fields. CNNs are suitable for processing Euclidean structure data with translation invariance such as image, text, speech, etc. This kind of data takes one of the data units as the central node and has the same number of neighbors, so the features of the data can be extracted sequentially by defining a globally shared convolution kernel. However, in the real world, the non-Euclidean structure data of knowledge graph, social network, chemical molecule and other graph structures are increasing, which is characterized by non-translation invariance, and the number of neighboring nodes may be different, so the convolution kernel cannot be used to sequentially extract the data information of the same structure. The application of CNNs on non-Euclidean structure data has limitations, so the Graph Convolutional Networks (GCNs) [2] were proposed to generalize CNNs to the above graph-structured data. GCNs and its variants are also widely used in many fields, such as traffic prediction [3], computer vision [4–6], machine translation [7, 8], disease prediction [9, 10], social analysis [11], recommendation system [12] and so on.

**Fig 1:**
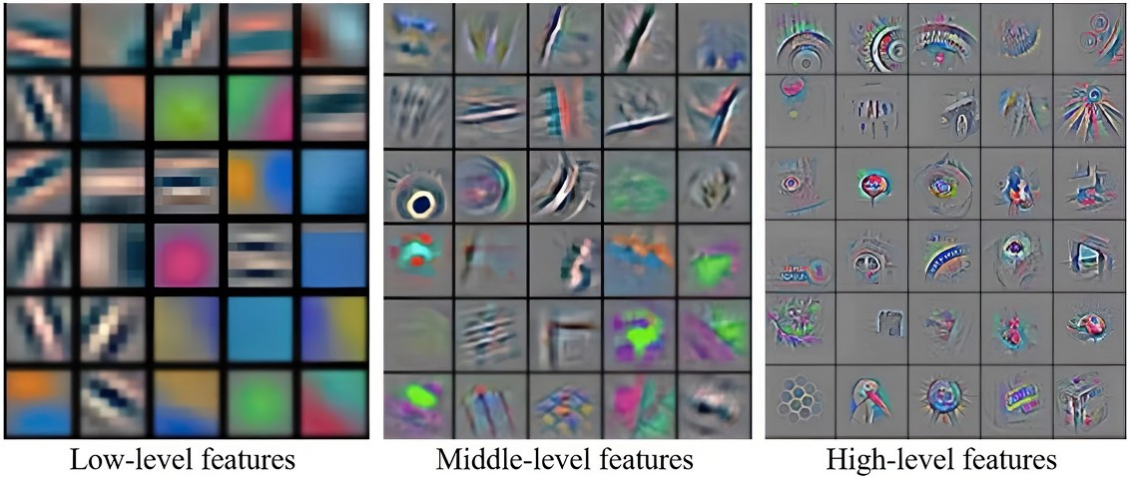
Object features extracted by neural networks with different depths [13].

In the field of image recognition, theoretically, the deeper the network, the more abstract high-level information can be extracted. The multi-layer network structure of the convolutional neural networks can learn the features of different levels of the image, and the extracted features are also richer. The shallow network has a small perception area and can learn the local regional features of the image, such as the edge and color of the object. The deep network has a larger perception area and can learn more abstract features of images, such as shape, contour, property, location and other high-dimensional features of objects. The ability of deep convolutional neural networks currently used in image recognition tasks to recognize objects even surpasses humans. As shown in Fig 1, the shallow network can only extract low-level features such as colors and lines, the intermediate network can extract middle-level features such as edges and contours, and the deep network can extract abstract high-level features such as shape, property, and category.

Therefore, we hope to introduce the concept of deep network in CNNs into GCNs, so that GCNs can also achieve the effect of deep CNNs, which can be used to extract high-order abstract features of nodes. However, most current state-of-the-art GCN models are no more than 3 to 4 layers deep and are usually limited to very shallow models. Q. Li et al. [14] pointed out that the shallow GCN model such as double-layer GCN [2] has its limitations and requires a large number of extra tags for model training. When only a few tags are given, the shallow GCN model is difficult to aggregate multi-hop neighborhoods information to the central node and is easily affected by the local properties of the convolution filter. Some papers [14–16] have analyzed the limitations of GCN, and pointed out that overlaying too many graph convolutional layers will lead to the over-smoothing problem, so that the features of nodes tend to be consistent, and different nodes will become indistinguishable. Xu et al. [17] studied the expressive ability of popular GNN variants and found that if GNN has strong expressive ability, different multiple aggregates must be synthesized into different representations. They also propose a theoretical framework to analyze the expressive ability of GNN to capture different graph structures, and show that popular GNN variants cannot learn to distinguish some simple graph structures. Alon et al. [18] pointed out that one of the main problems of training GNN is that it is difficult for them to propagate information between remote nodes in the graph. Each node in the graph has a very large number of K-order neighbors that when a remote node information is transmitted, it will be compressed / distorted. This phenomenon is called "over-squashing". Similar problems also appear in CNNs. Due to the chain rule, with the deepening of the network, the gradient may vanish or explode in the process of back propagation, and the deep neural network may degenerate into a shallow neural network, or even the network becomes unlearnable. To solve the problem of gradient vanishing / explosion in CNNs, He et al. [19] proposed the ResNet to make skip connections in some layers of the network, weaken the strong connection between each layer by adding residual connections and nonlinear transformation to achieve identity mapping, and get better results as the network deepens. On the basis of ResNet, Huang et al. [20] proposed DenseNet, which connects all layers to each other, realizes feature reuse, and makes it easier to achieve gradient back propagation and make the network easier to train.

In this paper, we hope to improve the performance of graph convolution network by deepening it, extract richer high-order abstract features of nodes by using deep network, and verify it on graph classification task. We use the ideas of ResNet and DenseNet in convolutional neural networks to conduct research on deep graph convolutional networks. In order to ensure that the deep network can at least achieve the performance of the shallow network, we transfer the shallow node features to the deep network, and reuse the features before each layer of the network. At the same time, the identity mapping mechanism is introduced into the linear transformation of node features. We show how to successfully integrate the concepts and methods of deep convolution neural network into a new general framework of graph neural network, and extensively analyze the accuracy and stability of the proposed deep graph convolutional network based on the new general framework. We show that GCN up to 32 layers can be successfully trained by using a non-local message passing framework, and we intend to test deeper networks in future research. Compared with 22 baseline methods such as 6 graph kernels and 16 other graph neural network methods, the proposed deep GCN model DGCNNII achieves first place in graph classification task on 4/5 bioinformatics datasets. Compared with DGCNN [21], when only the graph convolution part of the model is replaced, the accuracy of DGCNNII on the 5 datasets is about 3.96%–17.88% higher than that of DGCNN.

Our contributions in this paper are as follows. 1) We propose a novel general framework for graph neural networks called **Non-local Message Passing (NLMP)**. 2) Based on the NLMP framework, we propose a new spatial graph convolution layer to extract node multiscale high-order node features. 3) A novel end-to-end deep learning model DGCNNII for graph classification tasks is proposed. It can directly accept graphs as input without any pre-processing of the data. 4) Experimental results on benchmark graph classification datasets show that our **Deep Graph Convolutional Neural Network II (DGCNNII)** significantly outperforms graph kernel methods and numerous other deep learning methods on graph classification task.

## 2 Related works

### 2.1 Message Passing Neural Network (MPNN)

In recent years, the general framework of Message Passing Neural Network (MPNN) proposed by Gilmer et al. [22] has been applied to various graph related tasks, especially in graph supervised learning tasks. Such as SafeDrug, a drug recommendation model based on MPNN framework proposed by Yang et al. [23] which can encode the connectivity and function of drug molecules to achieve safe and accurate drug recommendation. Dasoulas et al. [24] proposed a graph neural network called Colored Local Iterative Procedure (CLIP) on the basis of MPNN, which used color to eliminate the ambiguity of the same node, and proved that this representation is a universal approximator of continuous functions on graphs with node properties, and demonstrate that this simple coloring scheme can theoretically and empirically improve the expressiveness of message passing neural networks. In terms of node update, the general framework of MPNN is mainly divided into two parts, one is to aggregate the information of neighboring nodes and edges around the vertex, and the other is to update the node information iteratively by fusing the vertex features of the previous iteration with the vertex features of the current aggregation. Specifically, see the following equations:

$$m_{i}^{\left(t+1\right)}=\underset{v_{j}\in N\left(v_{i}\right)}{\sum }M_{t}\left(h_{i}^{\left(t\right)},h_{j}^{\left(t\right)},e_{ij}\right)$$ (1)

$$h_{i}^{\left(t+1\right)}=U_{t}\left(h_{i}^{\left(t\right)},m_{i}^{\left(t+1\right)}\right)$$ (2)

*M* and *U* in the above Eqs (1) and (2) represent the message transfer function and the message update function, respectively. where *v*_*i*_ denotes vertex *i*, *N*(*v*_*i*_) denotes the first-order neighbor nodes of vertex *i*, *h*_*i*_ denotes the feature vector of vertex *i*, *e*_*ij*_ denotes the edge <*v*_*i*_, *v*_*j*_>, *m*_*i*_ denotes the feature vector of vertex *i* after aggregation operation, and *t* denotes the rounds of message propagation iterations.

Eq (1) represents the message passing aggregation operation, vertex *i* aggregates its feature vector $h_{i}^{\left(t\right)}$ at moment *t* with its first-order neighbor nodes feature vector $h_{N\left(v_{i}\right)}^{\left(t\right)}$ and the information of edges to obtain the node feature vector $m_{i}^{\left(t+1\right)}$ after aggregate operation. Eq (2) represents the node information update operation. The node feature vector $h_{i}^{\left(t\right)}$ at the previous moment and the node feature vector $m_{i}^{\left(t+1\right)}$ obtained by this iteration are aggregated through the update function *U* to update the node information.

The core of the MPNN framework is the message passing function and the message update function. Different improvements are made for these two parts. In principle, any graph neural network model can be designed based on this framework. From the perspective of message passing framework, many variants of graph neural network models have been proposed, and these models have achieved excellent results in node classification and graph classification tasks. Table 1 lists the message passing functions and message update functions of some classical graph neural network models.

**Table 1:** Different graph neural network models based on MPNN framework.

Model	Message Passing Function	Message Update Function
GCN [2]	${m^{\left(t+1\right)}=\tilde{D}}^{-1/2}\tilde{A}{\tilde{D}}^{-1/2}H^{\left(t\right)}W^{\left(t\right)}$	$H^{\left(t+1\right)}=ReLU(m^{\left(t+1\right)})$
GraphSAGE-mean [25]	$m^{\left(t+1\right)}=W^{\left(t\right)}\cdot MEAN(\{h_{i}^{\left(t\right)}\}\cup \{h_{j}^{\left(t\right)},v_{j}\in N(v_{i})\})$	$h_{i}^{\left(t+1\right)}=ReLU(m^{\left(t+1\right)})$
R-GCN [26]	$m^{\left(t+1\right)}=\sum_{r\in R}\sum_{j\in N_{i}^{\left(r\right)}}\frac{1}{c_{i,r}}W_{r}^{\left(t\right)}h_{j}^{\left(t\right)}+W_{0}^{\left(t\right)}h_{i}^{\left(t\right)}$	$h_{i}^{\left(t+1\right)}=ReLU(m^{\left(t+1\right)})$
DGCNN [21]	${m^{\left(t+1\right)}=\tilde{D}}^{-1}\tilde{A}H^{\left(t\right)}W^{\left(t\right)}$	*H*^(*t*+1)^ = *tanh*(*m*^(*t*+1)^)
DiffPool [27]	${m^{\left(t+1\right)}=\tilde{D}}^{-1/2}\tilde{A}{\tilde{D}}^{-1/2}H^{\left(t\right)}W^{\left(t\right)}$	*H*^(*t*+1)^ = *ReLU*(*m*^(*t*+1)^)
GIN [17]	$m^{\left(t+1\right)}=\left(1+\epsilon^{\left(t+1\right)}\right)\cdot h_{i}^{\left(t\right)}+\sum_{v_{j}\in N(v_{i})}h_{j}^{\left(t\right)}$	$h_{i}^{\left(t+1\right)}={MLP}^{\left(t+1\right)}(m^{\left(t+1\right)})$

### 2.2 Non-local Neural Network (NLNN)

X. Wang et al. [28] proposed a Non-local Neural Network (NLNN) model applied in the field of computer vision, which uses deep neural networks to capture remote dependencies. The idea of non-local operation is based on the generalization of non-local mean operation proposed by Buades et al. [29] and others. Its core idea is to perform weighted calculation on the features of all specific locations. The specific location can be the pixel coordinates of the spatial dimension or the time coordinate of the time dimension. When it is migrated to the graph structure data, the specific location can be replaced by a node. The deep graph convolution proposed in this paper can extend the specific position to multiple-hop neighborhood nodes after several iterations. For large-scale graphs, as long as the graph convolution layers are deep enough, the dependency of each node can be extended to the whole graph for capturing more comprehensive structural information of nodes, but need to solve the over-smoothing problem caused by multi-layer graph convolution, the solution will be proposed below.

In the paper of Buades et al. [29], given discrete noise image *v* = {*v*(*i*)|*i* ∈ *I*}, where *v*(*i*) represents the pixel value of pixel *i* and *I* represents all the pixels of the image. For pixel *i*, the estimated value *NL*[*v*](*i*) represents the weighted average of all pixels in the image, and the specific equation is as follows:

$$NL\left[v\right]\left(i\right)=\underset{j\in I}{\sum }w\left(i,j\right)v\left(j\right)$$ (3)

Where {*w*(*i*, *j*)|*j* ∈ *I*} represents the similarity weight between pixel *i* and pixel *j*, and satisfies 0 ≤ *w*(*i*, j) ≤ 1, ∑_*j*_ *w*(*i*, *j*) = 1. The estimated value of pixel *i* is represented by calculating the sum of products with similarity weight between pixel *i* and pixel *j*(*j* ∈ *I*). The similarity weight in this method is similar to the attention weight in the “Attention Mechanism”. Therefore, the NLNN framework can be regarded as the unification of various current self-attention-based methods [30–32]. In the field of graph convolution, analogous to the definition of Gaussian filtering Eq (4), the generalized node non-local feature aggregation operation is defined as Eq (5):

$$\text{GB}{\left[I\right]}_{p}=\underset{q\in S}{\sum }G_{\sigma }\left(\left|\left|p-q\right|\right|\right)I_{q}$$ (4)

$$h_{i}^{\left(t+1\right)}=\frac{1}{C\left(h\right)}\underset{\forall j}{\sum }f\left(h_{i}^{\left(t\right)},h_{j}^{\left(t\right)}\right)\cdot g\left(h_{j}^{\left(t\right)}\right)$$ (5)

In the above Eq (4), GB[*I*]_*p*_ represents the value of the pixel *p* after Gaussian blurring, *I*_*q*_ represents the value of pixel *q*, *S* represents the non-local adjacent region of pixel *p*, and *G*_*σ*_ represents Gaussian function, which is used to calculate the weight value. $h_{i}^{\left(t\right)}$ in the above Eq (5) represents the feature of the target node *i* at time *t*, $h_{j}^{\left(t\right)}$ represents the feature of all nodes *j* related to the target node *i* at time *t*, and $f(h_{i}^{\left(t\right)},h_{j}^{\left(t\right)})$ is used to calculate the attention coefficient between node *i* and node *j*, 1/*C*(*h*) is used to normalize the results, and $g(h_{j}^{\left(t\right)})$ represents the function that transforms the features of the input node *j*. The idea of NLNN framework is to aggregate all the node features related to the target node according to different attention weights to achieve the update of the target node features. When designing the model for different problems, function *f* and function *g* can be designed differently. The specific design of the model in this paper will be introduced in Section 3.

### 2.3 Graph classification

The framework and model proposed in this paper will be applied to the task of graph classification. Graph classification is also called graph property prediction, i.e., given a set of graphs, the goal is to learn the mapping relationship between the graph and the corresponding category label, and apply the trained model and parameters to the category prediction of unknown graphs. For example, in the field of chemical molecules, the structural of molecules can be seen as graph structure data, and the class prediction of molecular structure is used to determine the mutagenicity, toxicity, and anticancer activity of compound molecules [33, 34]. In molecular biology, by classifying and predicting the protein structure, we can judge whether the unknown protein is an enzyme, so as to determine whether the unknown protein has a therapeutic effect on a disease [35, 36]. The methods of graph classification mainly include methods based on graph kernels [37, 38], methods of graph similarity matching [39] and methods based on deep learning.

At present, the common steps for predicting graph properties based on deep learning are: 1) Firstly, the existing graph convolution method [2, 17, 21, 25–27] is used to extract node features, and the embedded representation of nodes is obtained. 2) Then aggregate the embedded representation of all the nodes in the graph to represent the graph embedded representation. 3) Finally, graph embedding representation is used to predict graph properties. For the first step, we can use one of the existing GCN methods to learn the node embedding representation. In the second step, we can use the readout function (pooling function) to read out the nodes in a certain order, and embed the nodes in a specific order as the graph embedding representation. In the last step, we classify the graph according to the embedding of each subgraph. Therefore, the research on graph property prediction using deep learning mainly focuses on the following two aspects. One is how to extract node features more accurately. Node representation learning is the precondition of graph representation learning. In recent years, a lot of work has been done on how to learn node embedding or graph embedding through various graph neural networks [40]. The second is how to design a readout function to perform a pooling operation on the graph. For isomorphic graphs, the readout function should be read out in a consistent order according to the role of nodes in the graph to ensure that the structural features of the graph do not change after the pooling operation.

For node representation learning, the node representation learning methods used in graph classification models such as EigenGCN [41], DGCNN [21], DiffPool [27], SAGPool [42] all adopt message passing GCN and its variants. For the graph pooling readout function, the feature representation of all nodes can be simply added or averaged as the feature representation of the graph, but this method will ignore many key nodes and structural information in the graph. Therefore, many more complex node readout methods have been proposed in recent years. For example, the pooling operation of the EigenGCN model proposed by [41] is divided into two parts: First, the large graph is divided into multiple sub-graphs, each sub-graph is pooled to get a super node, and multiple super nodes form a coarsening graph. The original graph signal is then converted into a graph signal defined on the coarsening graph using EigenPooling. The Graph U-Nets model with an encoder-decoder structure proposed by Gao & Ji [43] can perform graph pooling and unpooling operations on graphs.

### 2.4 Over-smoothing in node-property prediction

In the field of image recognition, the deepening of the network will lead to gradient vanishing or gradient explosion, and the problem of network degradation occurs. In the field of graph neural networks, the stacking of too many graph convolution layers will lead to over-smoothing of nodes. When multiple layers are stacked, the representation of all nodes tends to be consistent, and the local structural features of nodes are lost, which is the phenomenon of over-smoothing. Therefore, most of the graph convolution network models adopt shallow structure, which will greatly limit the ability of the network to obtain high-order neighborhoods information and structural features. The common graph convolution networks usually have 2–3 layers. Take the 2-layer graph convolution network of Eq (6) as an example [44]. The first layer graph convolution aggregates the information of the first-order neighbors around the node into itself and transforms it nonlinearly, while the second-layer graph convolution aggregates the information of the second-order neighbor nodes and classifies them.


$$Z_{GCN}=softmax\left(\hat{\tilde{A}}ReLU\left(\hat{\tilde{A}}XW_{0}\right)W_{1}\right)$$ (6)

In recent years, a lot of research has been devoted to solving the over-smoothing problem of graph convolutional networks. Q. Li et al. [14] proved that the graph convolution of the GCN model is a special form of Laplace smoothing, where the features of a node and its neighbors are updated to new node features after a weighted average, and firstly proposed that deep GCNs are easy to cause over-smoothing. Experiments show that node embedding has been mixed on a small data set with only 5 convolution layers. In order to solve the problem of node over-smoothing, Rong et al. [45] proposed DropEdge, whose idea is to randomly delete some edges of the input graph during model training, and DropEdge can be regarded as a message reducer to reduce the over-smoothing problem caused by multi-layer graph convolution to a certain extent. Feng et al. [46] proposed stochastic neural network architecture GRAND, which reduces the sensitivity of nodes to their neighbors by randomly discarding some nodes or some features of nodes. Although the above two methods can alleviate the over-smoothing phenomenon of deep graph convolution networks, but simply deleting nodes or edges will destroy the original data features and structural integrity. In addition to the above methods, there are some works as follows that try to do deep message passing by dealing with adjacency matrices.

#### 2.4.1 SGC

The SGC model proposed by F. Wu et al. [47] reduces the complexity of graph convolution computation by removing nonlinear transformations and folding weight matrices between continuous layers, by computing the graph convolution matrix to the K power in a single-layer neural network to capture higher-order information. Ordinary GCN convolution of each layer mainly includes three steps: node information propagation, node feature linear transformation and node nonlinear activation. For the classification task, if the Softmax function is finally used, the final classification result is Eq (7), and the specific process of graph convolution of each layer is Eq (8).

$${\hat{Y}}_{GCN}=softmax\left(\tilde{P}H^{K-1}W^{K}\right)$$ (7)

$$H^{\left(t+1\right)}=ReLU\left(\tilde{P}H^{\left(t\right)}W^{\left(t\right)}\right)$$ (8)

Where ${\tilde{P}=\tilde{D}}^{-1/2}\tilde{A}{\tilde{D}}^{-1/2}$ denotes the symmetric normalized adjacency matrix, 0 ≤ t ≤ K, and the input node features matrix *X* = *H*^(0)^.

SGC assumes that the nonlinear operation of node features is not important to GCN, and the effect of GCN mainly comes from the feature propagation between nodes. If the nonlinear transformation of each layer is deleted, it still has the same "receptive field" as K-layer GCN. Then the above Eq (7) is converted into the following Eq (9):

$${\hat{Y}}_{GCN}=softmax\left(\tilde{P}\ldots \tilde{P}\tilde{P}XW^{\left(1\right)}W^{\left(2\right)}\ldots W^{\left(K\right)}\right)$$ (9)

In order to simplify the above equation, the normalized adjacency matrix $\tilde{P}$ can be raised to the power of K, and the collapsed adjacency matrix ${\tilde{P}}^{K}$ can be obtained. At the same time, the product of multiple weight matrices can be replaced with one weight matrix, and the following simplified equation can be obtained:

$${\hat{Y}}_{GCN}=softmax\left({\tilde{P}}^{K}XW\right)$$ (10)

Through experiments, the effect of SGC is almost the same as that of GCN [2], and the speed is faster than GCN. But the premise of this method is to assume that the nonlinear transformation of node features is not important, which loses the powerful expressive ability of nonlinear structures.

#### 2.4.2 MAGNA

G. Wang et al. [48] aimed at the limitation that one-layer graph convolution in the existing GAT [31] model can only aggregate first-order neighbors information, allowing associations between nodes that are not directly connected in the network. Based on the powers of the 1-hop attention matrix *A*, the attention score of multi-hop neighborhoods is calculated through the following attention diffusion process:

$$\tilde{A}=\overset{\infty }{\underset{i=0}{\sum }}\theta_{i}A^{i}$$ (11)

Where *i* represents the number of paths from node *h* to node *t*, and the maximum length is *i*, thus increasing the receiving field of attention, *θ*_*i*_ represents attention attenuation factor, and satisfies $\sum_{i=0}^{\infty }\theta_{i}=1$, *θ*_*i*_ > 0, *θ*_*i*_ > *θ*_i+1_, i.e., the higher the order of attention factor, the smaller the weight. This method also assumes that there is a correlation between nodes that are not directly connected.

### 3 Deep Graph Convolutional Neural Network II (DGCNNII)

**Fig 2:**
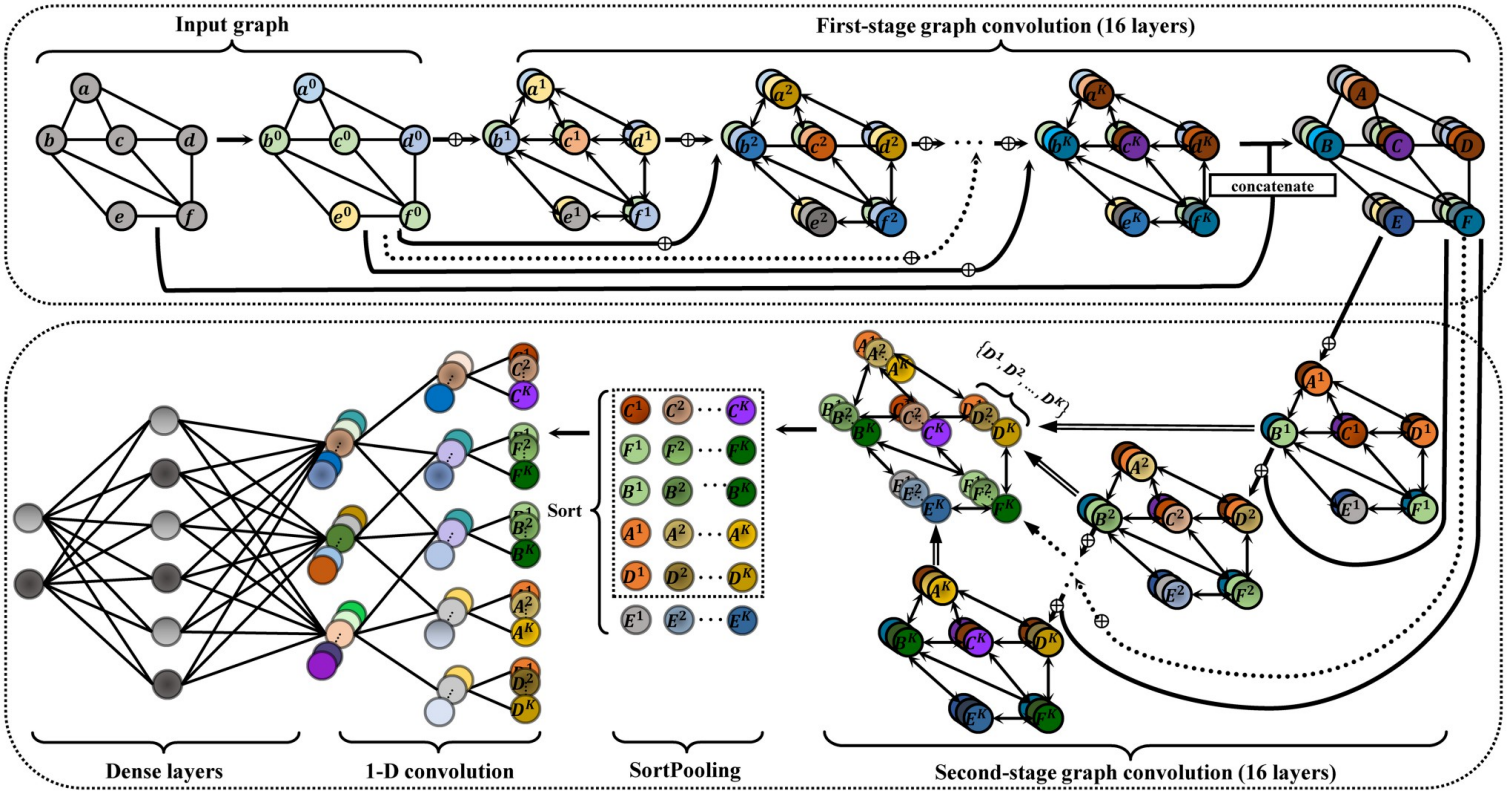
The overall structure of DGCNNII. The input graph with arbitrary structure first obtains the fine high-dimensional node features through the 16 layers of graph convolution in the first-stage, and then inputs them into the 16 layers of graph convolution in the second-stage, and the high-dimensional node features propagate deeply among the neighbors. Finally, the SortPooling layer is used to sort and intercept the nodes, and transfer them to the common convolution layer and dense layer for graph classification model training. Features are visualized as colors.

In this section, we propose DGCNNII for graph classification, which consists of four parts: 1) The graph convolution layers of the first-stage (16 layers) is used to extract the rich structure information of the input graph, and the rich high-dimensional features of the input nodes can be obtained. 2) The second-stage graph convolutional layers (16 layers) concatenates the high-dimensional node features extracted by the first-stage graph convolutional layers and the initial low-dimensional features as input to extract the deeper structural information and node features, then define a consistent vertex ordering. 3) The SortPooling layer sorts the node features output by the second-stage graph convolution layers, and unifies the number of nodes as the input of the next stage. 4) One-dimensional convolution layers and dense layers read the sorted continuous node features for graph attribute prediction. The following Fig 2 shows the architecture of DGCNNII. Table 2 summarizes the symbols used in this paper.

**Table 2:** Table of symbols used in this paper.

Symbol	Definition
*G* = (*V*, E)	*G*: input graph, *V*: node set, *E*: edge set.
|*V*| = *n*	*n*: number of nodes.
*v* ∈ *V*	nodes in *G*.
*x*_*v*_ ∈ *R*^*c*^	*x*_*v*_: node feature vector, *c*: feature dimension.
*X* ∈ *R*^*n×c*^	initial feature matrix.
$h_{i}^{k}$	node embedding of node *i* in the k^th^ layer.
*N*(*v*)	set of one-hop neighbors of node *v* in *G*.
*M*(·)	message aggregation function.
*H* ^*k*^	matrix of activations in the k^th^ layer.
*W* ^*k*^	a layer-specific trainable weight matrix.
$\tilde{A}$	adjacency matrix of the undirected graph *G* with added self-connections.
$\tilde{D}$	degree matrix of undirected graph *G*.
*σ*(·)	activation function.
*f*(*i*, *j*)	a function used to calculate the attention coefficient between i and j
*g*(·)	feature transformation function.
*δ*_*k*_	the weight matrix decay parameter of the k^th^ layer
*Z* ^*k*^	the k^th^ layer output of DGCNNII.
*A*_*GAT*_ ∈ *R*^*n*×*n*^	adjacency matrix based on node attention coefficients.
*α*, β, γ	hyper-parameters for adjusting the proportion of information aggregation.
*I*_*n*_	identity matrix.
*a*	the weight vector that projects the concatenate vector to the scalar

### 3.1 Non-local Message Passing Neural Network (NLMP)

Section 2 introduces two commonly used general frameworks of graph neural networks, namely MPNN and NLNN. The general framework of graph neural networks is to generalize and abstract the similar GNN networks structure, and integrate it into a unified framework, which provides ideas for flexible design and improvement of the model. The MPNN framework summarizes various GNN models and their variants from the perspective of message aggregation and updating. The NLNN framework is a generalized summary of the GNN model based on the attention mechanism. For details, see Sections 2.1 and 2.2.

Inspired by the successful application of deep neural networks in the image field, we aim to design a deep graph neural network framework which can solve the over-smoothing problem, in order to extract more remote dependencies of nodes. This framework can make the node information aggregation of graph neural network not only rely on local information, but also make vertices aggregate information from multi-hop neighborhoods by stacking multi-layer graph convolution. For graphs of different scales, the corresponding depth and information aggregation technology can be designed to aggregate the node information and structural information of the whole graph to the target node to extract higher-dimensional abstract features. The NLMP framework proposed in this paper is as follows:

$$h_{i}^{\left(t+1\right)}=\underset{v_{j}\in N\left(v_{i}\right)}{\sum }M_{t}\left(h_{i}^{\left(t\right)},h_{j}^{\left(t\right)},h_{i}^{\left(0\right)},h_{i}^{\left(t-1\right)},e_{ij}\right)$$ (12)

Compared with the Eq (1) of the MPNN framework, the above equation aggregates more information $h_{i}^{\left(0\right)}$ and $h_{i}^{\left(t-1\right)}$. For the aggregation update of target node *i*, it aggregates not only the information of its first-order neighboring nodes {$h_{j}^{\left(t\right)}|v_{j}\in N(v_{i})$} at time *t*, but also the initial input feature $h_{i}^{\left(0\right)}$ of node *i* and the node feature $h_{i}^{\left(t-1\right)}$ at the previous time. The method of aggregating $h_{i}^{\left(0\right)}$ and $h_{i}^{\left(t-1\right)}$ in the NLMP framework borrows the ideas from ResNet model and DenseNet model in the image field. The reason for introducing residual connections and dense connections in the process of node information aggregation is that when the graph neural network is deep enough, even if the node information goes through multiple rounds of iterations, some initial features $h_{i}^{\left(0\right)}$ of the nodes are still retained. Through back propagation learning, achieves at least the same effect as shallow networks, and the over-smoothing phenomenon is significantly reduced. The previous moment of node features $h_{i}^{\left(t-1\right)}$ is introduced, so that the result of each graph convolution layer always carries some node features generated by all previous layers, so that the final output node features of the model includes part of the output results of all convolutional layers. The dense connection in this paper does not connect the output results of all layers before each layer to itself, but only connects the results of the previous layer to itself, and the dependence of node features on the previous layer can be adjusted by setting parameters, it makes the adjustment of the model more flexible.

In order to investigate why graph neural networks can achieve good results, Q. Li et al. [14] compared GCNs with the simplest Fully Connected Networks (FCNs). The hierarchical propagation rules of conventional FCNs (Eq (13)) and the common neighboring information aggregation mode of GCN [2] (Eq (14)) are respectively as follows:

$$H^{\left(l+1\right)}=\;\sigma \left(H^{\left(l\right)}W^{\left(l\right)}\right)$$ (13)

$$H^{\left(l+1\right)}=\;\sigma \left({\tilde{D}}^{-\frac{1}{2}}\tilde{A}{\tilde{D}}^{-\frac{1}{2}}H^{\left(l\right)}W^{\left(l\right)}\right)$$ (14)

The only difference between GCN and FCN is that GCN multiplies the normalized adjacency matrix ${\tilde{D}}^{-1/2}\tilde{A}{\tilde{D}}^{-1/2}$ left by the feature matrix *H* and then right multiplies the weight matrix *W*. Therefore, the feature matrix processed by the normalized adjacency matrix is the reason for the good effect of graph convolution. The author also proves that graph convolution is a special form of Laplace smoothing, namely symmetric Laplace smoothing. Laplace smoothing averages the information of nodes and their neighbors as a new feature of nodes. Since the nodes in the same subgraph are often densely connected, after the import of deep graph convolution, the average aggregation of neighborhoods information makes the features of all nodes in the same subgraph tend to be consistent. And in the relational graph data, the influence of different types of neighbors on the target node is weakened. Therefore, using the idea of NLNN framework for reference, an adjacency matrix based on attention mechanism is introduced to aggregate neighbor nodes, which makes the NLMP framework proposed in this paper more generalized and scalable, easy adapts to multi-relational and single relational graph data, and makes up for the over-smoothing problem caused by average aggregation of neighbor nodes. The more specific NLMP framework design is as follows:

$$h_{i}^{\left(t+1\right)}=M_{t}\left\{\left[\frac{1}{C\left(h\right)}\underset{v_{j}\in N\left(v_{i}\right)}{\sum }f\left(h_{i}^{\left(t\right)},h_{j}^{\left(t\right)}\right)\cdot g\left(h_{j}^{\left(t\right)}\right)\right],\;g(h_{i}^{\left(0\right)}),g\left(h_{i}^{\left(t-1\right)}\right)\right\}$$ (15)

In the NLMP framework, *f* represents the Gaussian function that calculates the attention coefficients between nodes, *g* represents the node feature transformation function, *M* represents the message aggregation function, and the factor 1C(h) is used to normalize the results. The specific design of the above equation and the graph convolution layer used in this paper will be described in detail in Section 3.2.

### 3.2 Graph convolution layer

#### 3.2.1 Adjacency matrix based on attention coefficient

In the image field, the similarity between pixel *i* and pixel *j* can be defined as a decreasing function of the weighted Euclidean distance, ${\left||v\left(N_{i}\right)-v(N_{j})|\right|}_{2}^{2}$ [29]. We transfer the concept of similarity between pixels to graph data, compare the similarity between nodes can be converted to measure the similarity of node feature vectors, and the similarity can be calculated by the inner product of node vectors after linear transformation. According to the non-local mean operation [29] and the bilateral filter proposed by Tomasi & Manduchi [49], Gaussian function can be selected:

$$f\left(h_{i},h_{j}\right)=e^{\theta {\left(h_{i}\right)}^{T}\varphi \left(h_{j}\right)}$$ (16)

Where *θ*(*h*_*i*_) = *W*_*θ*_*h*_*i*_, *φ*(*h*_*j*_) = *W*_*φ*_*h*_*j*_. Based on the NLMP framework proposed in this paper, the Gaussian function used to calculate the attention coefficient between nodes adopts the concatenation function in the GAT model proposed by Velickovic et al. [31]:

$$f\left(h_{i},h_{j}\right)=e^{LeakyRelu(a^{T}[Wh_{i}||Wh_{j}])}$$ (17)

Where *h*_*i*_ and *h*_*j*_ represent the feature vector of node *i* and node *j* respectively, *h* ∈ *R*^*b*×1^ represents the feature vector, *W* ∈ *R*^*a*×*b*^ represents the learnable weight matrix, and || represents the concatenate operation. Therefore, (*Wh*_*i*_||*Wh*_*j*_) ∈ *R*^(2*a*×1)^. And *a*^*T*^ ∈ *R*^2*a*×1^ is the weight vector that projects the concatenate vector to the scalar. After regularizing the Gaussian function, the attention coefficient between node *i* and node *j* is obtained:

$$\frac{1}{C\left(h\right)}f\left(h_{i},h_{j}\right)=\frac{\text{exp}(LeakyRelu(a^{T}[Wh_{i}||Wh_{j}]))}{\sum_{k\in N_{i}}\text{exp}(LeakyRelu(a^{T}[Wh_{i}||Wh_{k}]))}$$ (18)

#### 3.2.2 Feature transformation based on identity mapping

For the feature transformation function *g* in Eq (15), we can use a simple linear transformation function *g*(*h*) = *Wh*, but Klicpera et al. [44] pointed out that frequent interactions between different dimensions of the feature matrix degrades the performance of the model. The GCNII model proposed by Chen et al. [50] borrows the idea of identity mapping in ResNet, and introduces the mechanism of identity mapping into GCN. The identity matrix *I*_*n*_ is added to the weight matrix *W*, and the weight of the identity matrix increases with the number of layers. The goal is to ensure that the deep GCN model achieves at least the same performance as the shallow GCN. Therefore, the linear transformation function in this paper is set as:

$$g\left(h\right)=\left(\left(1-\delta_{l}\right)I_{n}+\delta_{l}W^{\left(l\right)}\right)h$$ (19)

In the above equation, *δ*_*l*_ is the weight matrix decay parameter that changes with the number of layers *l*. We refer to the setting δl=log(λl+1) in GCNII, and *λ* is the hyper-parameter. *δ*_*l*_ makes the weight matrix adaptively decay as the number of layer increases.

#### 3.2.3 Proposed form of graph convolution

In conclusion, given an input graph *G* = (*V*, *E*) and its node feature matrix *X* ∈ *R*^*n*×*c*^, the proposed DGCNNII in this paper extracts the non-local structure information of nodes by stacking deep graph convolutions to achieve the aggregation of non-local neighborhood nodes information. We define the (*t* + 1)^*th*^ graph convolutional layer as:

$$Z^{\left(t+1\right)}=\sigma \left(\left(\alpha A_{GAT}^{\left(t\right)}Z^{\left(t\right)}+\beta Z^{\left(t-1\right)}+\gamma X\right)\left(\left(1-\delta^{\left(t\right)}\right)I_{n}+\delta^{\left(t\right)}W^{\left(t\right)}\right)\right)$$ (20)

In the above equation, *Z*^(*t*)^ is the output result of the t^th^ graph convolution layer. Initially can make *Z*^(0)^ = *Z*^(1)^ = *X*, $A_{GAT}^{\left(t\right)}\in R^{n\times n}$ is the adjacency matrix based on the attention coefficient of the t^th^ graph convolution layer of the input graph *G*, where ${\left(A_{GAT}^{\left(t\right)}\right)}_{ij}\in \{0,r\}$. If there is an edge between node *i* and node *j*, namely (*v*_*i*_, *v*_*j*_) ∈ *E*, then ${\left(A_{GAT}^{\left(t\right)}\right)}_{ij}=r$, the real number *r* ∈ (0,1) represents the attention coefficient of the correlation between the two nodes, otherwise ${\left(A_{GAT}^{\left(t\right)}\right)}_{ij}=0$. details as follows:

$${\left(A_{GAT}^{\left(t\right)}\right)}_{ij}=\frac{\text{exp}(LeakyRelu({\left(a^{\left(t\right)}\right)}^{T}[W_{GAT}^{\left(t\right)}h_{i}^{\left(t\right)}||W_{GAT}^{\left(t\right)}h_{j}^{\left(t\right)}]))}{\sum_{k\in N_{i}^{\left(t\right)}}\;\text{exp}(LeakyRelu({\left(a^{\left(t\right)}\right)}^{T}[W_{GAT}^{\left(t\right)}h_{i}^{\left(t\right)}||W_{GAT}^{\left(t\right)}h_{k}^{\left(t\right)}]))}$$ (21)

Each layer of graph convolution is divided into the following four steps: 1) Firstly, the node features after nonlinear activation are propagated through *A*_*GAT*_*Z* to the neighboring nodes and the nodes themselves according to different attention weights to obtain the new node feature matrix *Y* = *A*_*GAT*_*Z*. 2) The new feature matrix $\overset{-}{Y}$ is obtained by summing *Y*, the result of the graph convolution of the previous layer, and the initial node feature matrix according to the corresponding proportion. 3) Through $\overset{-}{Y}(\left(1-\delta \right)I_{n}+\delta W)$, the node feature matrix is transformed by linear feature transformation based on identity mapping. 4) Finally, the nonlinear activation function is applied to the result of the previous step, and the graph convolution result is output. Repeat the above four steps for each layer of GCN.

We suggest that the initial feature matrix *X* in the Eq (20) can be an one-hot vector, or it can transform the feature dimension through a fully connected neural network according to the needs of model. $Z^{\left(t-1\right)}\in R^{n\times c_{t-1}}$ is the output of the (*t* − 1)^*th*^ graph convolutional layer, *c*_*t*−1_ is the number of output feature channels of the node in the (*t* − 1)^*th*^ layer, The weight matrix $W^{\left(t\right)}\in R^{c_{t}\times c_{t+1}}$ is used to map the *c*_*t*_ node feature channels into *c*_*t*+1_ feature channels. *β* and *γ* are parameters that control the proportion of the previous layer output and initial node features, which can be adjusted manually. *α* can be simply set to *α* = 1 − *β* − *γ*. In this paper, both *β* and *γ* are set to 0.1, and *σ* is the nonlinear activation function. After the second-stage graph convolution of the model is completed, a layer is added to connect the output result *Z*^(*t*)^ (*t* = 1, …, *K*) of each graph convolution layer, denoted as *Z*^1:*K*^ = [*Z*^(1)^, *Z*^(2)^, …, *Z*^(*K*)^], each row of the concatenated output *Z*^1:*K*^ can be viewed as a "multi-scale feature vector" of a vertex and used as input to the remaining layers.

### 3.3 Remaining layers

#### 3.3.1 Graph pooling layer

The graph pooling layer is used to aggregate the node features learned by the graph convolution layer for subsequent operations. Common graph pooling methods in graph neural network models include: TopKPooling, SAGPooling, Set2Set, etc. Table 3 lists some other commonly used graph pooling methods and specific operations. $x_{k}^{\left(i\right)}$ represents the i^th^ dimension of node embedding, *N*_*i*_ represents the neighbor nodes of node *i*.

**Table 3:** Common graph pooling methods.

Graph pooling methods	Graph pooling equation
SumPooling	$r^{\left(i\right)}=\sum_{k=1}^{N_{i}}x_{k}^{\left(i\right)}$
AvgPooling	$r^{\left(i\right)}=\frac{1}{N_{i}}\sum_{k=1}^{N_{i}}x_{k}^{\left(i\right)}$
MaxPooling [51]	$r^{\left(i\right)}={max}_{k=1}^{N_{i}}(x_{k}^{\left(i\right)})$
GlobalAttentionPooling [52]	$r^{\left(i\right)}=\sum_{k=1}^{N_{i}}softmax(f_{gate}(x^{\left(i\right)}k))f_{feat}(x_{k}^{\left(i\right)})$

In order to compare with the DGCNN framework of Zhang et al. [21], and to reflect the advantages of Deep Graph Convolution Neural Network II (DGCNNII) proposed in this paper, the layers behind the graph convolution layers adopt the same configuration as DGCNN. For details, please refer to the papers of Zhang et al. The graph pooling of this paper uses SortPooling. As mentioned by Zhang et al., the output of the graph convolution layer in this paper is also a continuous WL (Weisfeiler-Lehman) color, and the deeper the convolution layer is, the more *Z*^(*K*)^ can divide the node into different colors / groups. The DGCNN model has only 4 layers of graph convolution, and the DGCNNII model proposed in this paper has a depth of 32 layers. The nodes are sorted in descending order according to the last channel *Z*^(*K*)^ output by the graph convolution layer. If the values of two nodes are the same in the *Z*^(*K*)^ channel, they are compared through the previous layer *Z*^(*K*−1)^, and so on. The output of the graph convolution layers is a tensor of shape $n\times \sum_{1}^{K}c_{t}$ as the input of the SortPooling layer, because the number of nodes in each subgraph is different, in order to facilitate the subsequent processing of the network, the SortPooning layer truncates / expands the input tensor of $n\times \sum_{1}^{K}c_{t}$ to the tensor of $k\times \sum_{1}^{K}c_{t}$ size.

#### 3.3.2 Traditional layers

Consistent with the paper of Zhang et al., first convert the $k\times \sum_{1}^{K}c_{t}$ tensor output by the SortPooling layer into a row vector of $k(\sum_{1}^{K}c_{t})\times 1$, and then add maximum pooling layers, one-dimensional convolution layers, fully connected layers, and a softmax layer, etc.

## 4 Discussion

As mentioned earlier, we need the graph convolution layers deep enough to divide the nodes into different groups / categories as much as possible. However, when the graph convolutional layers are stacked too much, there will be serious over-smoothing problem. In a densely connected graph, each node has many common neighbor nodes, if there are too many layers in the graph convolutional neural network, the number of aggregated neighbor nodes will increase and the number of overlapping nodes will increase, which will easily lead to the consistency of the final node feature representation, and the features of different nodes will be covered up.

The DGCNNII model proposed on the basis of the NLMP framework provides a solution to the above problems and achieves state-of-the-art results. This framework mainly solves the problem that the node features tend to be consistent caused by the average aggregation of a large number of overlapping neighbor nodes. The graph convolution in DGCNNII does not simply use the normalized adjacency matrix to aggregate neighbor information according to the degree of nodes, but introduces the graph attention mechanism to aggregate information according to the different attention coefficients between nodes and their neighbors to adaptively learn the weights of nodes with different importance to avoid average aggregation leading to the consistency of node features. At the same time, residual connections and dense connections are introduced to make each information aggregation includes the initial features of nodes and the results of the previous graph convolution layer, ensuring that the model achieves at least the results of the shallow version. In order to better eliminate the over-smoothing phenomenon, the identity mapping mechanism is also introduced. DGCNNII’s double-stage graph convolution framework can extract rich node features for deep node information propagation, combined with the above techniques can achieve amazing results.

## 5 Experiments

In this section, we use 5 bioinformatics datasets to validate the performance of DGCNNII on graph classification task. And compared with the classical graph kernel methods and other deep learning methods. In order to reflect the effect of DGCNNII on eliminating the over-smoothing phenomenon, we quantify the graph smoothness of each graph convolutional layer of the model and compare it with DGCNN. Ablation studies were also carried out to demonstrate the effectiveness of the model architecture. Finally, we analyze the model based on the experimental results. All experiments run on a computer with 12-core Intel(R) i7-12700KF CPU, 16 GB RAM, and NVIDIA Geforce RTX 3080 12GB GPU. We use Pytorch to implement our methods.

### 5.1 Experimental setup

#### 5.1.1 Dataset

Because DGCNNII has the advantage of extracting rich node information based on non-local structural features, this paper uses 5 bioinformatic datasets with node labels, named MUTAG [53], PTC [54], PROTEINS [55], D&D [56], NCI1 [57], instead of using purely structural datasets without node labels, the initial node features of each bioinformatics dataset are represented by one-hot vectors.

1. The MUTAG dataset consists of 188 compounds, which are divided into two categories according to their mutagenic effect on bacteria. Each compound represents a graph, and each atom represents the nodes in the subgraph.
2. The PTC dataset also consists of 344 compounds (graphs), which are divided into two categories according to whether they are carcinogenic to mice. Compounds are composed of 19 kinds of atoms (nodes).
3. The PROTEINS dataset consists of 1113 protein structure graphs, all protein structures (graphs) are divided into two classes of enzymes/non-enzymes, and nodes consist of 3 classes.
4. The D&D dataset consists of 1178 protein structure graphs, all protein structures (graphs) are classified into enzymatic/non-enzymatic categories, and the nodes are composed of 82 amino acids.
5. NCI1 is a cancer cell activity screening compound dataset, and the graph labels are divided into two categories with/without anticancer activity.

The following table summarizes the specific parameters of the above datasets.

#### 5.1.2 Model settings

In order to mainly compare the DGCNN model and highlight the advantages of the deep graph convolution proposed in this paper, except for the graph convolution layers, the experimental settings of other parts of the model refer to DGCNN. In order to achieve a fair comparison, we follow to set up the experiments for 10 times and report the average test accuracy using the 10-fold cross validation. The graph convolution of DGCNNII has two stages, and each stage has 16 graph convolution layers. The first 15 layers of graph convolution in the first-stage all have 128 output channels, and the number of output channels of the 16^th^ layer of graph convolution is set differently according to different data sets. In this paper, the number of output channels (output feature dimension) of the 16^th^ layer is set to 32, 64, 128, 32, 128 for the MUTAG, PTC, NCI1, PROTEINS, D&D datasets, respectively. The output feature matrix of the 16^th^ layer of DGCNNII for each of the above datasets also contains the initial one-hot vector of the node. The operation of concatenating the initial feature vector of nodes after the output of the first-stage graph convolution can effectively reduce the phenomenon of node over-smoothing. The quantitative measurement of the graph smoothness of each layer of the model in Section 5.3 can show the effectiveness of concatenating operation. In particular, there is a fully connected layer that converts the initial input one-hot vector into 128 dimensions before the first-stage of graph convolution, which is used for feature dimension conversion. The second-stage graph convolution consists of a fully connected layer which converts the input feature dimension into 128 dimensions and 16 graph convolution layers. The first 15 layers of graph convolution have 128 output channels, and the last layer convolution is a single channel output. We use the one-channel features output from the last graph convolutional layer for node sorting. The remaining layers are composed of two one-dimensional convolution layers and one dense layer. The configurations of the two one-dimensional convolution layers are: 1) Filter size is 2, maximum pooling layer with step size 2, output channel is 16. 2) Filter size is 5, step size is 1, output channel is 32. The dense layer has 128 hidden units, and finally the Softmax layer.

#### 5.1.3 Parameter settings

The *α*, β and γ in Eq (20) are set to 0.8, 0.1 and 0.1 respectively. The *λ* in the weight matrix attenuation parameter $log(\frac{\lambda }{l}+1)$ is set to 0.5. A dropout layer with dropout rate 0.5 is used after the dense layer. The training batch size is set to 1, and the partition ratio of the training sets and the test sets is 9:1. Rectified linear units (ReLU) is used as a nonlinear function in the convolution layer and other layers. Stochastic gradient descent (SGD) with the ADAM updating rule [58] was used for optimization. In order to fairly compare the performance of DGCNN and DGCNNII in the graph classification task, and highlight the improvement brought by the use of deep graph convolutional layers, we use the same learning rate and epoch number as DGCNN in the MUTAG, PTC, NCI1, PROTEINS, D&D dataset without hyper-parameter adjustment.

### 5.2 Comparison with other graph classification methods

#### 5.2.1 Comparison with graph kernel

We compare DGCNNII with the following 6 classical graph kernel methods: Shortest-Path Kernel [59], the Graphlet Kernel [60], the Random Walk Kernel [61], the Weisfeiler-Lehman subtree kernel [62], the Propagation Kernel [63] and Deep Graph Kernels [64]. For fair comparison, we use a single network structure on all datasets. We compare the results of DGCNNII with methods based on graph kernel mentioned above and take the reported accuracy directly from their papers (“–” means not available). The results are shown in Table 4, and the best results are shown in bold.

**Table 4:** Comparison of graph classification accuracy with graph kernel methods.

Methods	Dataset				
	MUTAG	PTC	NCI1	PROTEINS	D&D
SP	87.28±0.55	58.24±2.44	73.47±0.11	75.07±0.54	78.86± 0.26
GK	81.39±1.74	55.65±0.46	62.49±0.27	71.39±0.31	74.38±0.69
RW	79.17±2.07	55.91±0.32	>3 days	59.57±0.09	>3 days
WL	84.11±1.91	57.97±2.49	**84.46±0.45**	74.68±0.49	78.34±0.62
PK	76.00±2.69	59.50±2.44	82.54±0.47	73.68±0.68	78.25±0.51
DGK	87.44±2.72	60.08±2.55	80.31±0.46	75.68±0.54	-
DGCNNII (proposed)	**94.44**	**76.47±2.94**	80.32±0.76	**82.88±0.83**	**83.33±1.29**

From the results in Table 4, we can see that although DGCNNII uses the same single structure on all data sets, but still achieves excellent results compared with the kernel results. The accuracy on MUTAG, PTC, PROTEINS, and D&D are higher than that of all graph kernel baseline methods, and DGCNNII has achieved a significant improvement on MUTAG and PTC datasets with smaller graph sizes. It shows that the two small-scale datasets after 32-layer depth graph convolution, the nodes aggregate more comprehensive non-local information, which greatly improves the prediction accuracy.

#### 5.2.2 Comparison with other graph neural network models

We compare DGCNNII with a variety of graph classification baseline methods based on deep learning in recent years, including various models based on MPNN and NLNN framework. Among them, Diffusion-CNN (DCNN) [65] and PATCHY-SAN [66] both extend convolutional neural networks (CNNs) to general graph structure data for graph convolution operation. The convolution operation of ECC [67] and the conventional two-dimensional image convolution are both weighted average operation, but ECC can be applied to any graph structure, and the weight is determined by the edge weight between nodes. One-head graph attention network (1-head GAT) [31] can assign different weights to different nodes in the neighborhoods, rather than simply weighted average neighbor nodes. GCAPS-CNN is a graph capsule network proposed by Verma & Zhang [68]. AWE [69] proposes two anonymous sequence graph representation methods based on feature vector and embedding vector. S2S-N2N-PP [70] generates the node sequence of the graph through methods such as random walk, breadth first search, and shortest path, and then feeds it into a long short-term memory (LSTM) autoencoder for training to obtain a vector representation. The Motif-based filtering in the NEST [71] model can capture the fine structures in the network, while the convolution based on filtering embedding enables it to fully explore complex substructures and their combinations, thus carrying out graph classification tasks. CapsGNN [72] proposes a new vector propagation mode and hierarchical prediction, which makes the network more interpretable. GIN [17] proposes a simple architecture and has the same powerful graph isomorphism recognition capability as the Weisfeiler-Lehman graph kernel. MA-GCNN [73] is a new Motif-based attention graph convolutional neural network that can learn more discriminative and richer graph features. This paper also compares the baseline methods of four improved graph pooling functions, such as DiffPool [27], gPool [43], EigenPool [41] and SAGPool [42]. The specific comparison results are shown in Table 5. The best results are shown in bold.

**Table 5:** Comparison of graph classification accuracy with other graph neural network based methods.

Methods	Dataset				
	MUTAG	PTC	NCI1	PROTEINS	D&D
DCNN			56.61±1.04	61.29±1.60	58.09±0.53
PATCHY-SAN	92.63±4.21	62.29±5.68	76.34±1.68	75.00±2.51	76.27±2.64
ECC	89.44	-	76.82	-	72.54
GAT(1-head)	81.0	57.0	74.3	72.5	-
GCAPS-CNN	-	66.01±5.91	82.72±2.38	76.40±4.17	77.62±4.99
AWE	87.87±9.76	-	-	-	71.51±4.02
S2S-N2N-PP	89.86±1.10	64.54±1.10	**83.72±0.40**	76.61±0.50	-
NEST	91.85±1.57	67.42±1.83	81.59±0.46	76.54±0.26	78.11±0.36
CapsGNN	86.67±6.88	-	78.35±1.55	76.28±3.63	75.38±4.17
GIN	89.40±5.60	64.60±7.00	82.70±1.70	76.20±2.80	-
MA-GCNN	93.89±5.24	71.76±6.33	81.77±2.36	79.35±1.74	81.48±1.03
DiffPool	-	-	-	76.25	80.64
gPool	-	-	-	77.68	82.43
EigenPool	-	-	77.00	76.60	78.60
SAGPool	-	-	67.45±1.11	71.86±0.97	76.45±0.97
DGCNN	85.83±1.66	58.59±2.47	74.44±0.47	75.54±0.94	79.37±0.94
DGCNNII (proposed)	**94.44**	**76.47±2.94**	80.32±0.76	**82.88±0.83**	**83.33±1.29**

We can see that DGCNNII using a single structure surpasses all the baseline methods in terms of accuracy with only one exception on NCI1. It is worth mentioning that since MUTAG has only 188 graphs, under the 9:1 training / test set division, the test set has only 18 graphs. When the categories of 17 graphs are correctly predicted, the accuracy rate reaches 94.44%. However, in the actual prediction process of DGCNNII, a large number of cross-validation reached 100% accuracy, and it can be seen from Fig 5 that when the training reached the 20^th^ epoch, it had reached 100% accuracy, and continued to stabilize at more than 94%, while maintaining lower loss and higher AUC than DGCNN. DGCNNII also achieves the best results on PTC, with an average accuracy rate of 4.71%-19.47% higher than other baseline methods. Although not achieving the best accuracy on NCI1, but the accuracy rate is about 6% higher than DGCNN. While all other baseline methods have not reached 80% accuracy on PROTEINS dataset, DGCNNII achieves 81.08% accuracy. The highest accuracy is also achieved on the D&D dataset.

We analyze the NCI1 dataset. According to Table 6, we can see that although the NCI1 dataset has the largest number of subgraphs, but it is the only dataset with a higher number of node labels than the average number of subgraph nodes in the 5 datasets, which means that many subgraphs in NCI1 do not fully contain all types of nodes. While our proposed deep graph convolutional model uses the strategy of node information aggregation and update, although the deep network model can aggregate higher-order node information, due to the lack of information in the subgraphs of the dataset, all node features are not covered during training, so it cannot achieve the best results on the test set. We also compare and analyze the S2S-N2N-PP model which has the best classification result on NCI1. The method is to generate sequences from graphs by random walk, breadth-first search and shortest path, and then use recurrent neural network automatic encoder to embed graph sequences into continuous vector space to learn graph representation. S2S-N2N-PP does not adopt the information aggregation and update strategy of traditional GCN, and does not depend on the comprehensiveness of the training set, so it has achieved good results on the NCI1 data set.

**Table 6:** Common datasets for graph classification tasks.

Dataset	Graphs	Classes	Nodes(max)	Nodes(avg.)	Node Labels
MUTAG	188	2	28	17.93	7
PTC	344	2	109	25.56	19
NCI1	4110	2	111	29.87	37
PROTEINS	1113	2	620	39.06	3
D&D	1178	2	5748	284.32	82

### 5.3 Quantitative analysis the smoothness of graph nodes representations

**Fig 3:**
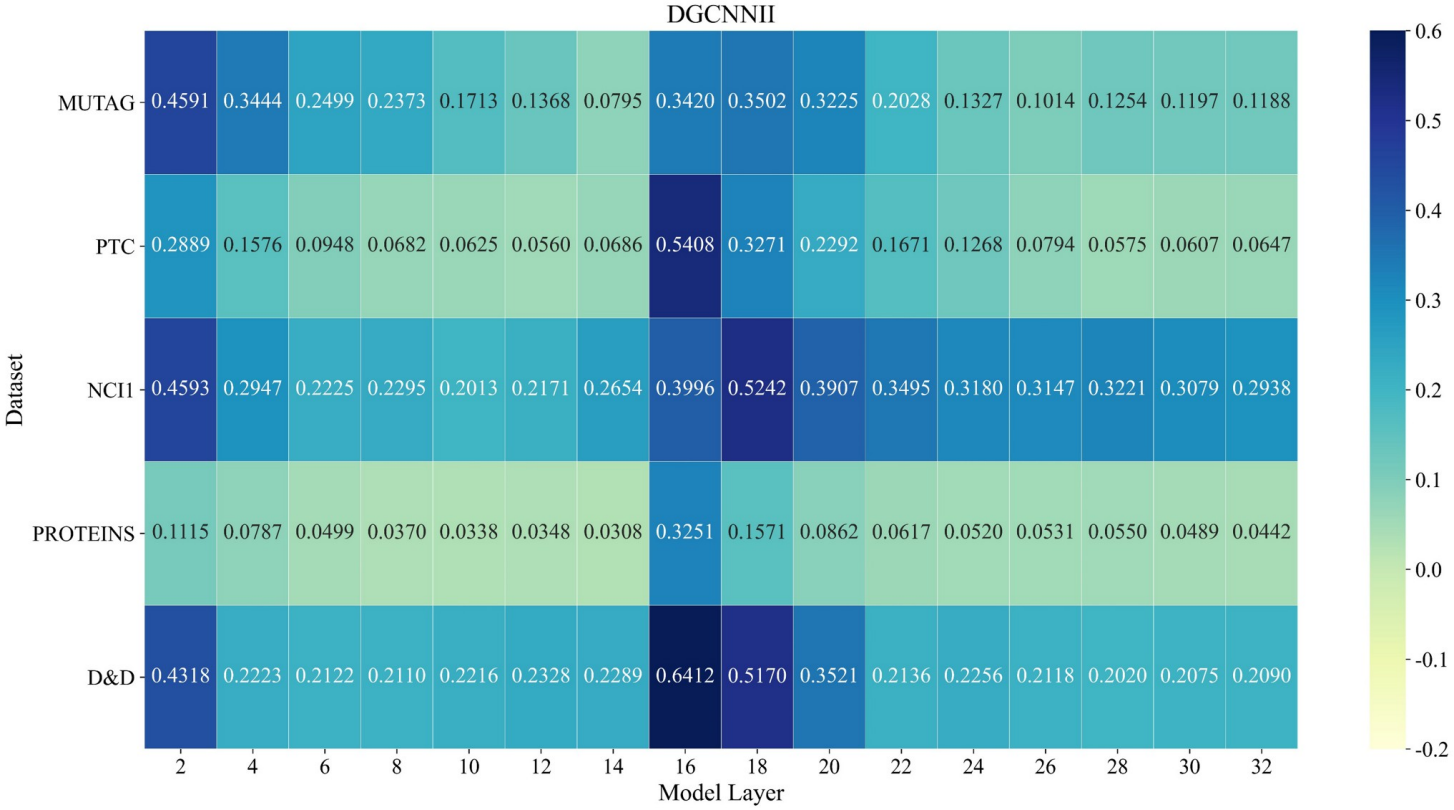
MAD values of different layers of DGCNNII on 5 datasets. Darker color means larger MAD value. We can find that at the output of the first-stage of DGCNNII (layer 16), the MAD value is greatly improved.

**Fig 4:**
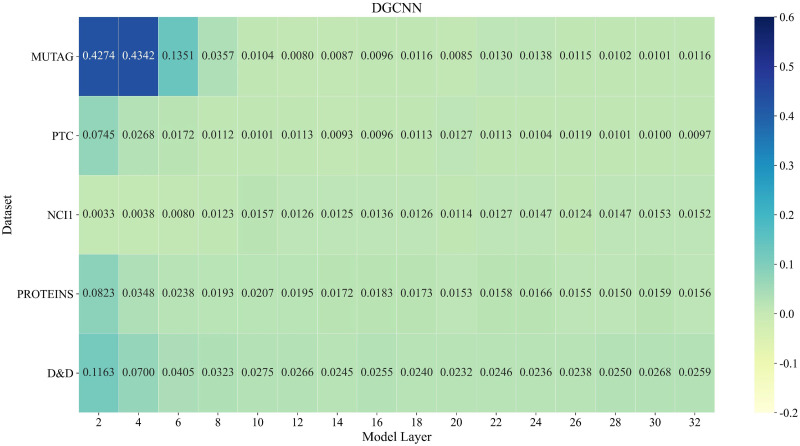
MAD values of different layers of 32-layer DGCNN on 5 datasets. Lighter color means smaller MAD value. We can find that the smoothness of the graph representation increases with the number of model layers, and the over-smoothing phenomenon of DGCNN is significantly more serious than that of DGCNNII.

In order to solve the problem of node over-smoothing caused by stacking too many layers of GCN, we redefine the widely used message passing neural network (MPNN) framework and propose a new non-local message passing framework (NLMP). Based on NLMP, a 32-layer deep graph convolutional neural network model DGCNNII is designed for graph classification tasks. This section will use a quantitative method to measure the graph smoothness of each layer of the model, and the quantitative method used is the quantitative metric Mean Average Distance (MAD) proposed by Chen et al. [74]. We deepen the DGCNN model from 4 layers to 32 layers, and compare the graph smoothness between the DGCNN and the 32-layer DGCNNII proposed in this paper. Through the experimental results of Figs 3 and 4, we can see that the MAD value of each layer of the DGCNNII model is significantly higher than that of the DGCNN model (the higher the MAD value is, the lower the smoothness of the graph representation is). It is quantitatively proved that the method proposed in this paper can effectively eliminate the over-smoothing problem caused by the deepening of GCN.

#### 5.3.1 MAD: Metric for smoothness

MAD reflects the smoothness of graph representation by computing the average of the average distances from nodes to other nodes in the graph. Since graph smoothness refers to the similarity of graph node representation, so the node similarity is measured by calculating the average distance between nodes. The equation for calculating the MAD value of the target node pair is:

$$MAD^{tgt}=\frac{\sum_{i=0}^{n}{\bar{D}}_{i}^{tgt}}{\sum_{i=0}^{n}1\left({\bar{D}}_{i}^{tgt}\right)}$$ (22)

Where 1(x) = 1 if x > 0 otherwise 0, that is, the MAD values of the target node pairs are the average of the non-zero values in ${\overset{-}{D}}_{i}^{tgt}$. The equation of ${\overset{-}{D}}_{i}^{tgt}$ and its parameters is:

$${\bar{D}}_{i}^{tgt}=\frac{\sum_{j=0}^{n}D_{ij}^{tgt}}{\sum_{j=0}^{n}1\left(D_{ij}^{tgt}\right)}$$ (23)

$$D^{tgt}=D○M^{tgt}$$ (24)

$$D_{ij}=1-\frac{H_{i,:}\cdot H_{j,:}}{\left|H_{i,:}\right|\cdot \left|H_{j,:}\right|}\;\;i,j\in \left[1,2,\ldots ,n\right]$$ (25)

The above Eq (23) means to average the non-zero elements in each row of *D*^*tgt*^. *M*^*tgt*^ ∈ *R*^*n*×*n*^ in Eq (24) represents the mask matrix with the same shape as the adjacency matrix, and ○ represents filtering the distance matrix *D* ∈ *R*^*n*×*n*^ by element-wise multiplication. Eq (25) represents the elements in *D*, *D* is obtained by calculating the cosine value between each node pairs, and *H* ∈ *R*^*n*×*h*^ is the graph representation matrix, where *n* is the number of nodes in the graph, term *h* is the hidden size. *H*_*k*,:_ represents the k^th^ row of *H*. We take the output of the last layer of the model as *H*.

It should be noted here that in the paper of Chen [74] et al., all node pairs in the graph are considered to calculate the MAD value, that is, *M*^*tgt*^ ∈ *R*^*n*×*n*^ is an all-one matrix. We consider that the diagonal matrix represents the node pair between the node and itself, and due to the characteristics of some data sets, the adjacent nodes are similar. So in the process of calculating the MAD value, the diagonal of *M*^*tgt*^ and the position of the adjacent nodes are all set 0, the rest of the positions are set to 1 to calculate the local smoothness of the graph representation. We use the same computational criteria in all experiments without affecting the objective comparison of experiments. Fig 3 shows the MAD values of different layers of our proposed 32-layer DGCNNII model on 5 datasets, and Fig 4 shows the MAD values of the deepened 32-layer DGCNN model on 5 datasets on different layers. The darker color in the figures means a larger MAD value, which means less similarity between the nodes in the graph, otherwise the more similar.

By quantitatively comparing the smoothness of each layer of the 32-layer DGCNNII and DGCNN model, we find that the smoothness of the graph representation of DGCNNII at the 32^nd^ layer is even lower than that of the 2–4 layer of DGCNN, which indicates that the deep graph convolution network construction method proposed by us can effectively reduce the phenomenon of over-smoothing. The subsequent ablation studies in this paper will also prove that the smoothness of graph representation is not the decisive factor affecting the result of graph classification task, the structure and depth of graph convolution network model can also affect the result of graph classification.

### 5.4 Ablation study

We will conduct ablation studies on the necessity of the double-stage structure of the DGCNNII and why the model chooses 32 layers.

#### 5.4.1 The necessity of double-stage model structure

In order to verify the function of the 16 layers of the second stage, we delete the last 16 layers of the DGCNNII model in Fig 2, leaving only the first 16 layers of the first stage. The graph classification results of the deleted model on the 5 data sets are shown in the first row "Only first stage (16layers)" of Table 7. In order to verify the function of 16 layers in the first stage, we change the double-stage model structure of DGCNNII into a 32-layer single stage model structure, and the graph classification results on 5 data sets are shown in the second row "Only one stage (32layers)" of Table 7. By comparing the experimental results of the above two modified models with the double-stage structure DGCNNII (32layers) model proposed in this paper, we can see that the result of the double-stage structure model is better than that of the single-layer structure model, so it is necessary to apply the double-stage structure model of DGCNNII.

**Table 7:** Experimental results of ablation study of DGCNNII model.

Methods (DGCNNII)	Dataset				
	MUTAG	PTC	NCI1	PROTEINS	D&D
Only first stage(16 layers)	92.11±0.87	74.76±2.51	78.35±0.76	81.08±0.54	82.05±0.80
Only one stage(32 layers)	92.11±0.91	70.82±2.57	75.91±0.76	80.18±0.81	82.05±0.83
DGCNNII-18(16+2 layers)	84.21±0.85	70.71±2.64	76.64±0.75	80.18±0.79	81.20±0.82
DGCNNII-22(16+6 layers)	92.11±0.91	68.88±2.54	78.59±0.75	79.28±0.79	81.20±0.82
DGCNNII-26(16+10 layers)	89.47±0.89	72.71±1.63	77.37±0.74	78.38±0.78	82.05±0.83
DGCNNII-30(16+14 layers)	94.44	74.59±2.66	79.32±0.79	81.98±0.82	82.91±0.84
DGCNNII(32 layers)	**94.44**	**76.47±2.94**	**80.32±0.76**	**82.88±0.83**	**83.33±1.29**

#### 5.4.2 Comparison of models with different layers

In order to compare the graph classification results of DGCNNII model with different layers, we carried out ablation studies for different layers of the model. Through Fig 3, we can see that the node feature of the first stage output of DGCNNII (layer 16) has a very low smoothness (higher MAD value), and through Table 7, we can also see that the DGCNNII with only first stage has a good graph classification accuracy. Therefore, we decided to adjust the number of layers in the second-stage to carry out the ablation study on the basis of retaining the first-stage. The specific experimental results are shown in Table 7 below. For example, "DGCNNII18 (16+2layers)" means adding 2 layers of second-stage graph convolution to the 16-layer of first stage, and "DGCNNII (32layers)" represents the 32-layer deep graph convolutional model (Fig 2) finally adopted in this paper. The experimental results show that the 32-layer DGCNNII achieves better results than the shallow version.

### 5.5 Comparison and analysis with DGCNN

In this paper, we propose a Non-local Message Passing (NLMP) framework, and a novel deep graph convolution layer and DGCNNII model based on NLMP are proposed. The DGCNNII model replaces the graph convolution part with our proposed deep graph convolution on the basis of the DGCNN model. The latter graph pooling layers and traditional layers are consistent with DGCNN. The purpose is to validate the advantages of the NLMP framework by comparing the performance of the two models on the graph classification task. Next, we analyze the performance of DGCNNII and DGCNN on the same dataset.

#### 5.5.1 Classification accuracy comparison

**Fig 5:**
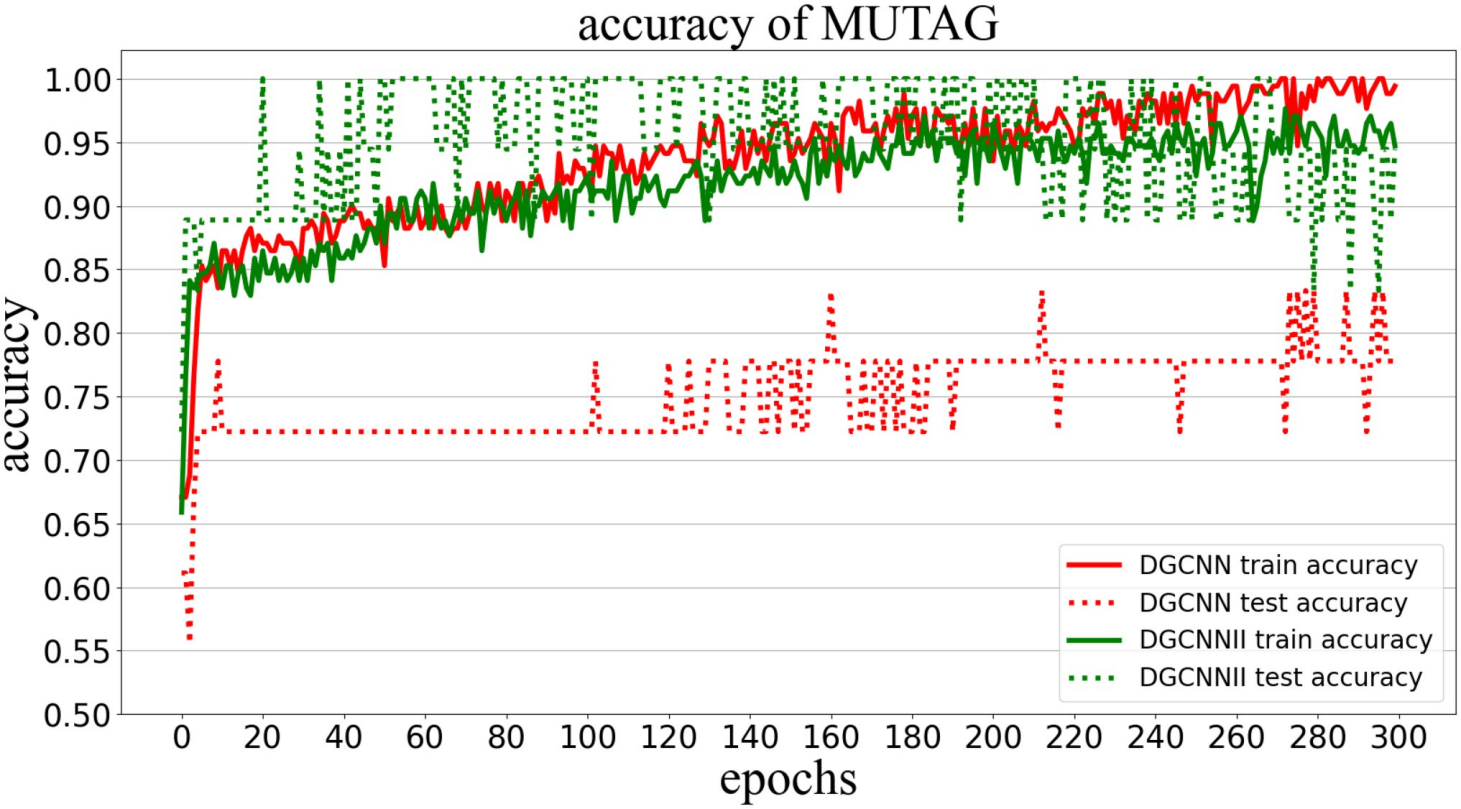
Comparison of graph classification accuracy between DGCNNII and DGCNN on MUTAG dataset.

**Fig 6:**
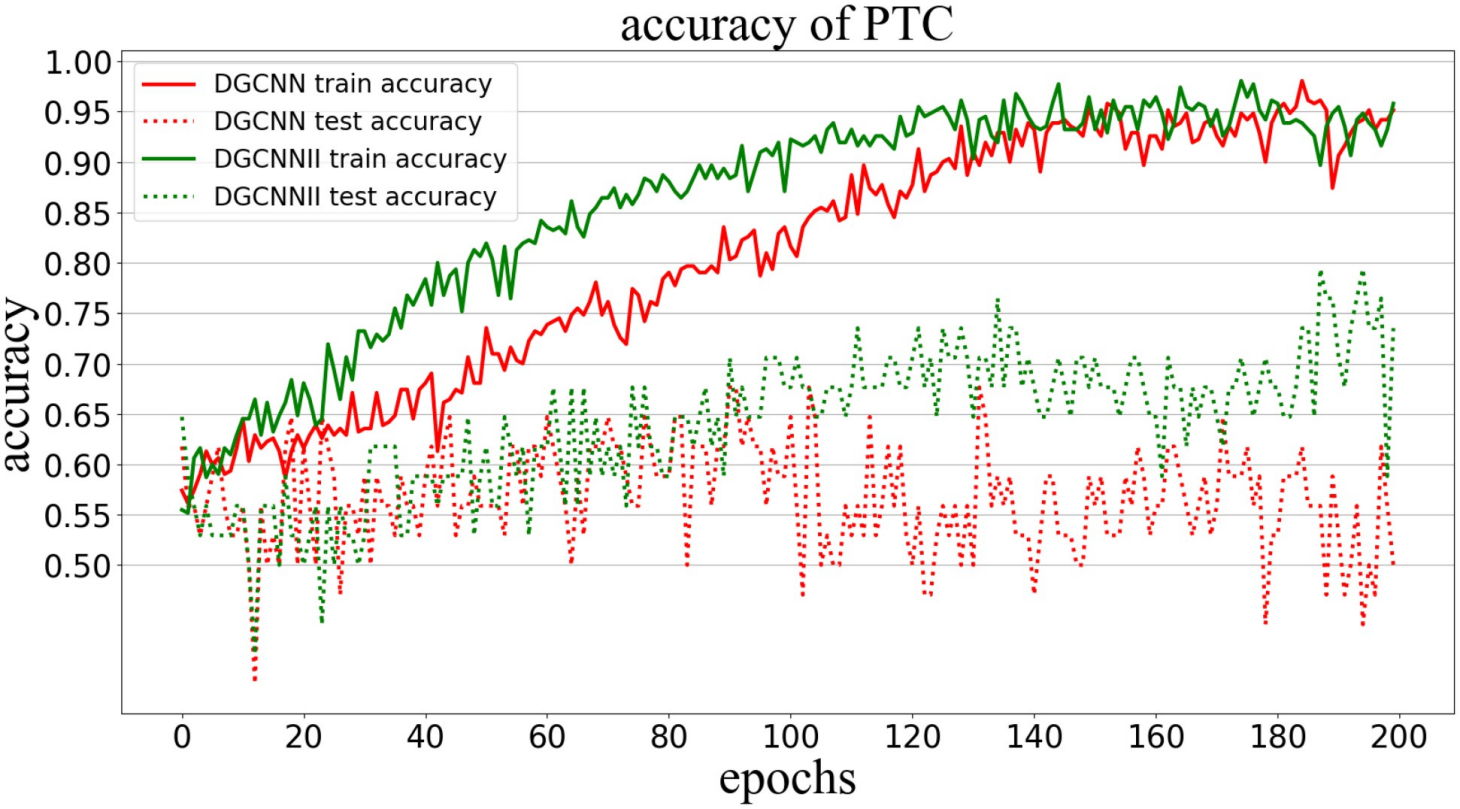
Comparison of graph classification accuracy between DGCNNII and DGCNN on PTC dataset.

**Fig 7:**
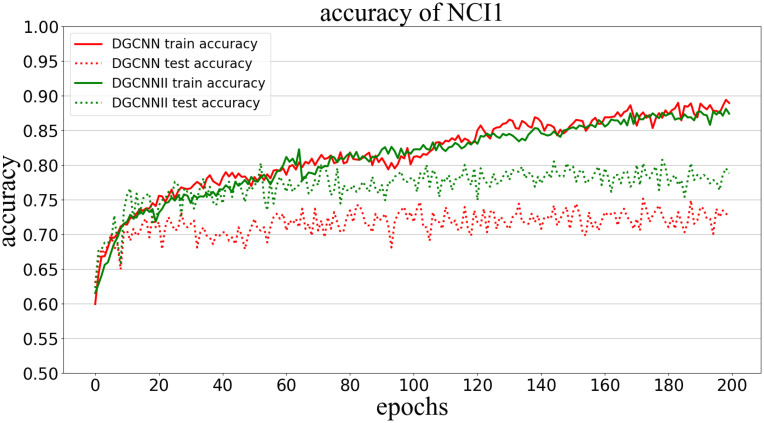
Comparison of graph classification accuracy between DGCNNII and DGCNN on NCI1 dataset.

**Fig 8:**
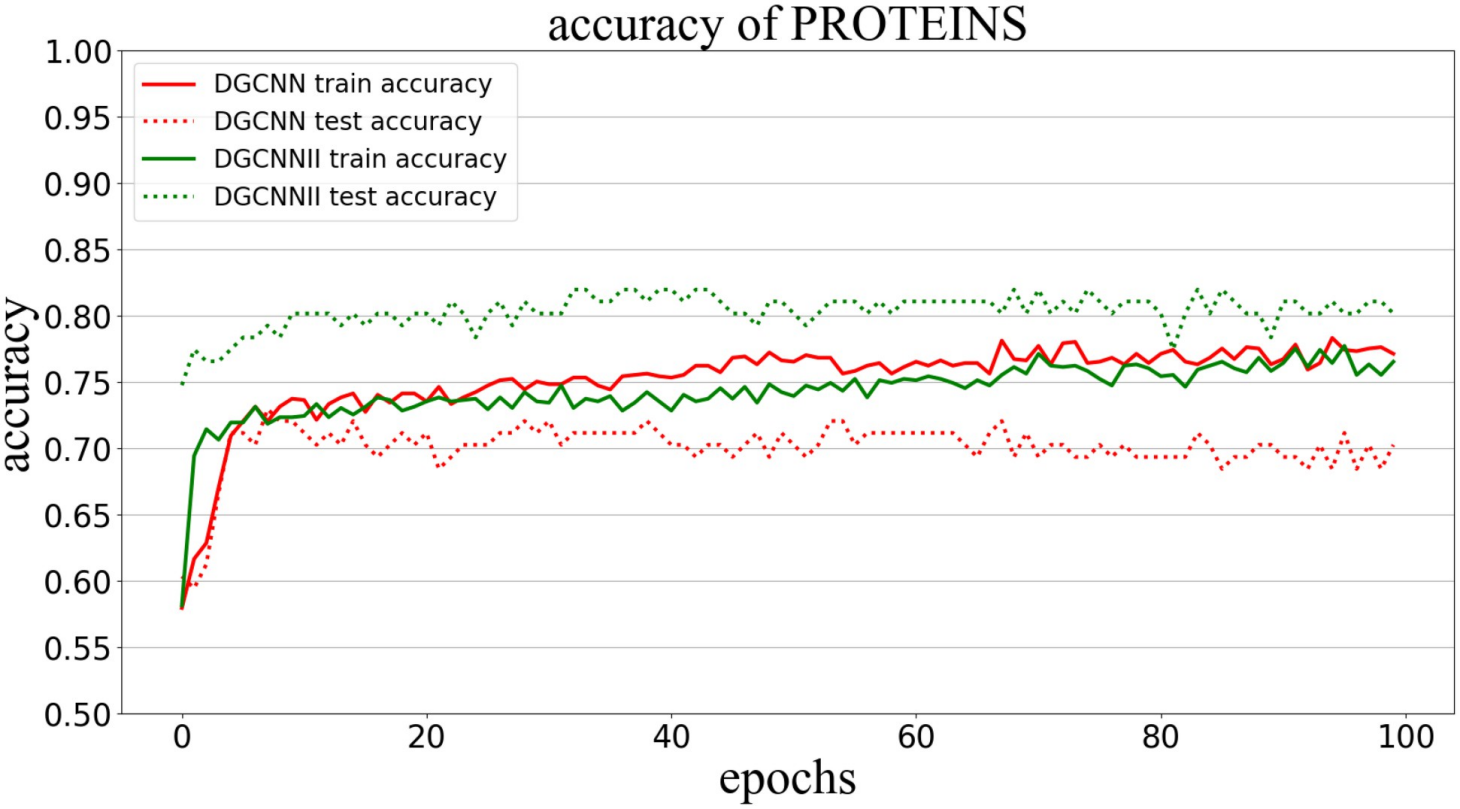
Comparison of graph classification accuracy between DGCNNII and DGCNN on PROTEINS dataset.

**Fig 9:**
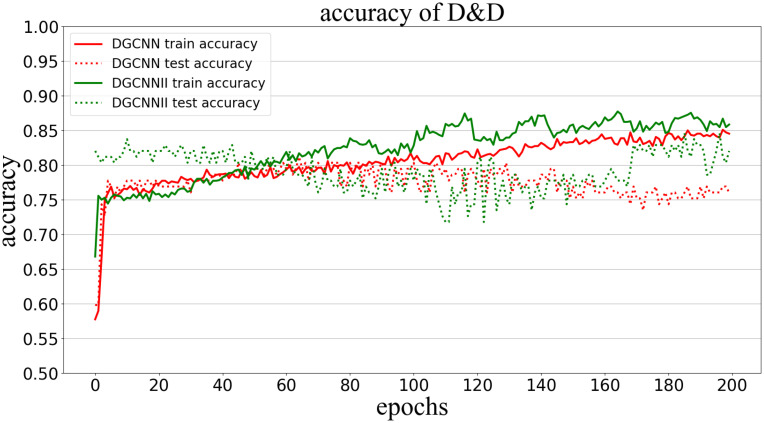
Comparison of graph classification accuracy between DGCNNII and DGCNN on D&D dataset.

Classification accuracy (Accuracy) is used to measure the proportion of correctly classified graphs in all graphs. The following Figs 5–9 show the classification accuracy curves of DGCNNII and DGCNN on 5 datasets with the change of training epochs.

As can be seen from the Fig 5, on the MUTAG data set, after the 20^th^ round of DGCNNII training, the accuracy of the test set can reach 100%, and then remain between 94% and 100%. However, DGCNN can only achieve the highest accuracy of about 85% in the 160^th^ round of training, and the accuracy of the test set of DGCNN is far lower than the accuracy of the training set, indicating that the DGCNN model has the phenomenon of over-fitting. However, from the accuracy curve of DGCNNII training set and the accuracy curve of test set in Fig 5, we can see that the difference of accuracy between test set and training set in the later epochs are very small, indicating that DGCNNII greatly **reduces the over-fitting phenomenon**.

As can be seen from the Fig 6, on the PTC data set, although the accuracy of the training set of the two models has been increasing, but the accuracy of the test set of DGCNN decreased after 100 epochs of training, while the accuracy of DGCNNII has been steadily increasing, indicating that DGCNNII is **more stable**.

As can be seen from the Fig 7, on the NCI1 data set, the accuracy of the training / test set of the two models has been steadily increasing. Although the accuracy of the training set of the two models is almost the same, but the accuracy of the test set of DGCNNII is always higher than that of DGCNN.

On the PROTEINS data set, it can be seen that the accuracy of the test set of DGCNNII is continuously stable and much higher than that of DGCNN.

On the D&D data set, the accuracy of the test set of DGCNN continues to decrease after the 140^th^ epoch of training, while the accuracy of the training set continues to increase, indicating that over-fitting occurred, but the accuracy of the test set of DGCNNII continues to increase, its stability and the ability to reduce over-fitting are further verified.

#### 5.5.2 Comparison of generalization ability

The generalization ability of the model in deep learning refers to the adaptability of the model to unknown samples, which can be measured by the classification accuracy of the test set. However, it should be noted that comparing the generalization ability of the two models needs to meet the following three conditions: 1) Both models are trained in the same training set. 2) The test set is unknown sample data. 3) The test set and the training set belong to the same distribution of data. All the experiments in this paper are carried out under the condition of meeting the above three conditions, so we can directly compare the generalization ability of the two models by comparing the graph classification accuracy of DGCNNII and DGCNN on 5 test data sets. As can be seen from Figs 5–9, the accuracy of DGCNNII on the 5 test data sets is higher than that of DGCNN, so DGCNNII has **stronger generalization ability** than DGCNN.

In the field of GNNs, with the stacking of a large number of graph convolution layers, there will be serious over-smoothing problem. This section makes an experimental comparison between the DGCNN model with 4 graph convolution layers and the DGCNNII model with 32 graph convolution layers. It can be clearly seen that the DGCNNII model based on the NLMP framework not only solves the problem of over-smoothing, but also exerts the ability of deep learning to extract abstract features, reduces the phenomenon of over-fitting, and maintains the accuracy higher than the general baseline method. It also has strong stability and generalization ability.

#### 5.5.3 Compare Area Under ROC Curve (AUC) and loss

**Fig 10:**
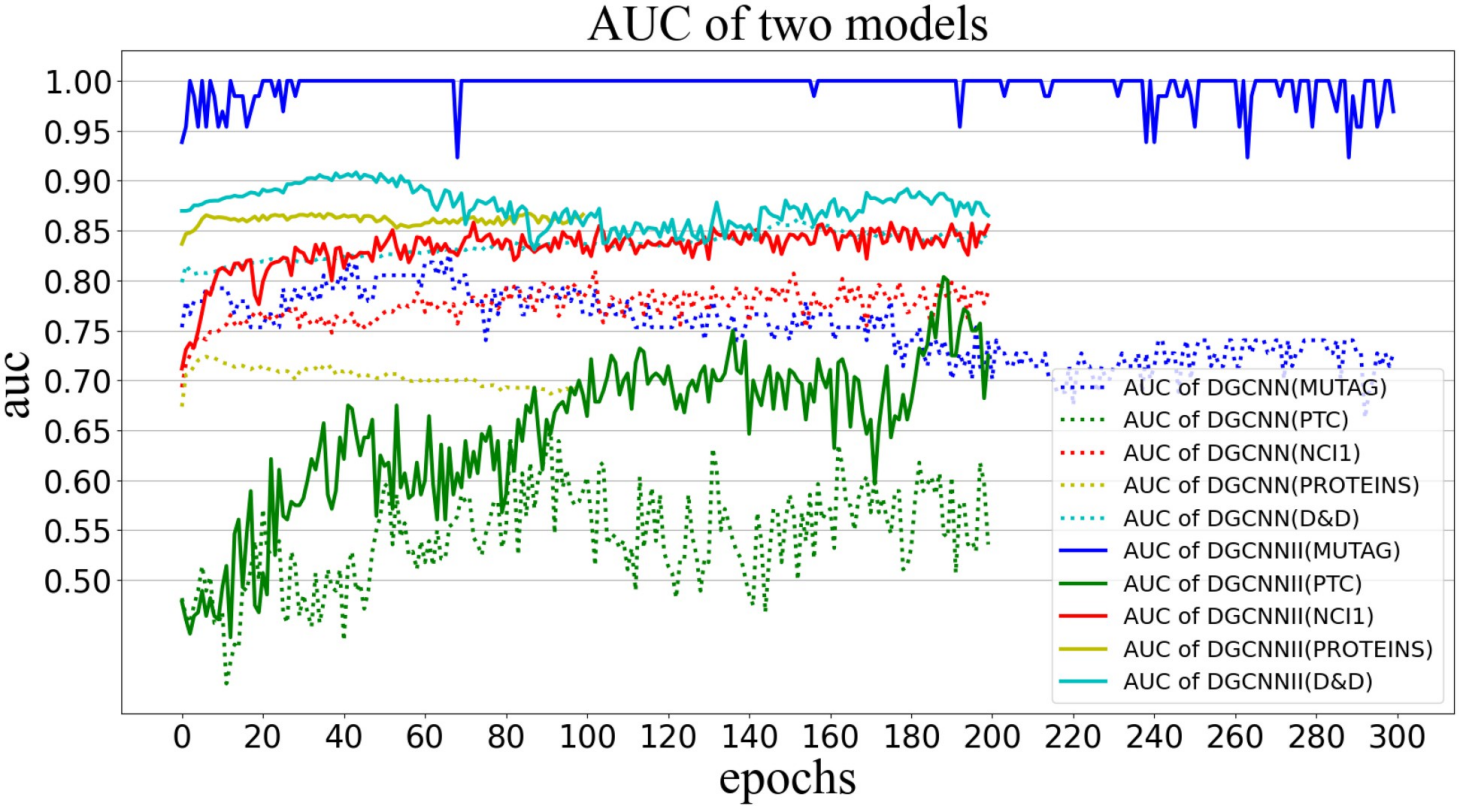
AUC values of DGCNNII and DGCNN on 5 test sets.

**Fig 11:**
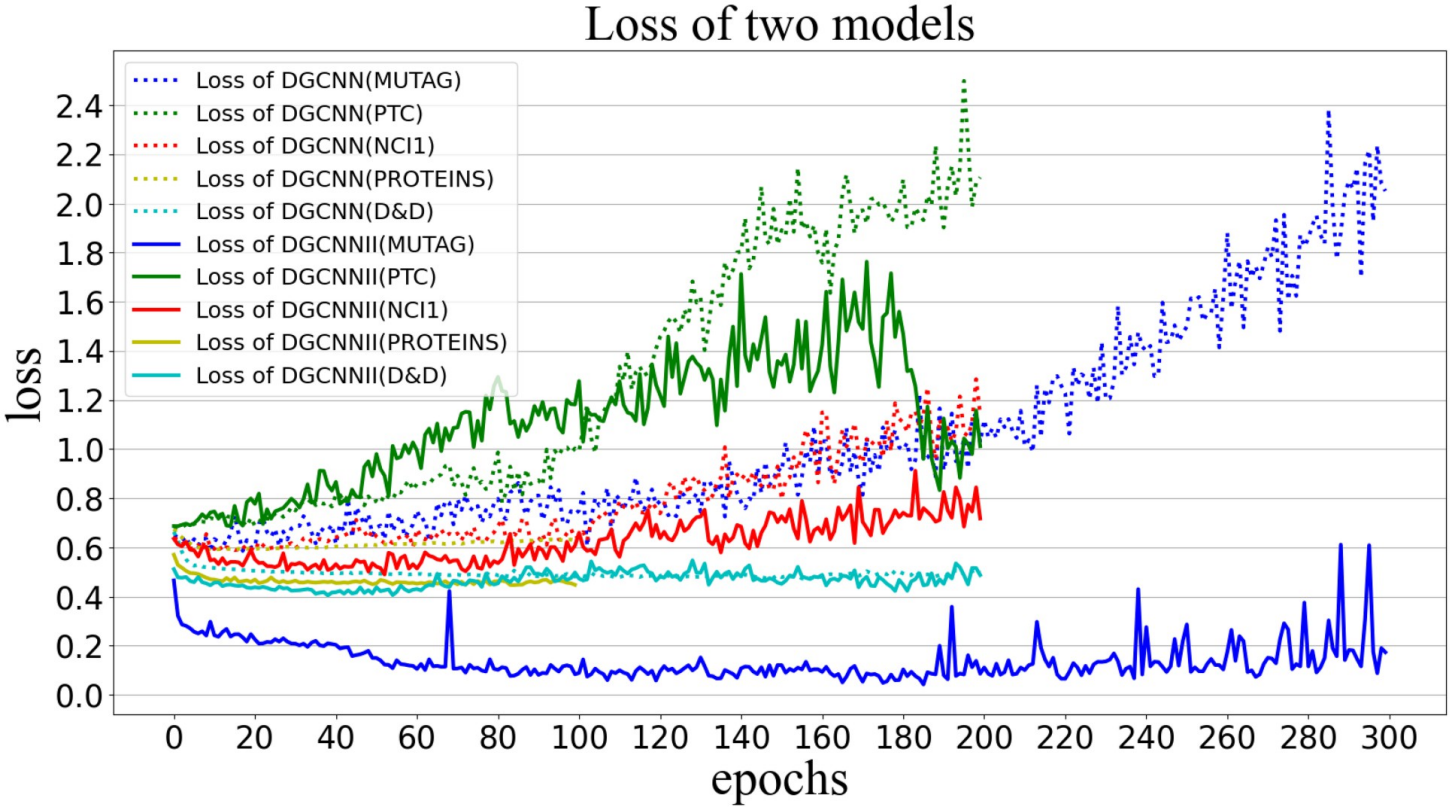
Loss values of DGCNNII and DGCNN on 5 test sets.

AUC can be used as an indicator to measure the quality of the classifier. When the AUC value is [0.5, 0.7], it means that it has low accuracy, and when the value is [0.7, 0.9], it has credible accuracy, when the value is greater than 0.9, the classifier has high accuracy, when the AUC value is less than 0.5, the model has no classification significance. Fig 10 visualizes the AUC values obtained by DGCNNII and DGCNN in each epoch on the 5 test sets. The dotted line represents the AUC value of the DGCNN model on the 5 datasets, the solid line represents the AUC value of the DGCNNII model, and the lines of different colors represent different datasets. MUTAG, PTC, NCI1, PROTEINS and D&D were trained for 300, 200, 200, 100, 200 epochs respectively. As can be seen from the Fig 10, the AUC values of all DGCNNII are higher than the corresponding DGCNN on each dataset. DGCNNII has high confidence with AUC values above 0.84 on all datasets except PTC, in particular, the AUC values of the MUTAG dataset basically reach 1.0. However, the AUC values of DGCNN on all datasets are lower than 0.84, and the AUC values of PTC are basically between 0.5–0.6, with relatively low confidence. But the AUC value of PTC data set on DGCNNII can reach 0.7–0.8, achieving reliable accuracy. The following Fig 11 shows the loss values of DGCNNII and DGCNN in the training process on 5 test sets. It can be seen that the loss values of DGCNNII on each data set are lower than those of DGCNN.

## 6 Conclusions

This paper proposes a novel graph neural network framework, named Non-local Message Passing (NLMP) framework. Compared with existing graph neural network frameworks, NLMP has many advantages. First, based on the NLMP framework, a deep graph neural network can be constructed to extract high-order neighbor nodes features almost without over-smoothing. Secondly, the neighbor aggregation scheme based on node attention weight and the graph convolution layer based on NLMP framework can extract the finer features of the nodes, highlight the key node information, and avoid the nodes over-smoothing. At the same time, various new message passing methods and attention mechanisms can be introduced based on this framework, making the model design more flexible and extensible. For the scalability of the model, the appropriate model depth can be selected by quantifying the smoothness of each layer of the DGCNNII. The ablation study in this paper also proves the effectiveness of the double-stage model structure, so we can flexibly combine and design models with different depths and layers for different data sets. The designed deep graph convolutional layer receives the original graph as input and outputs the feature matrix of the graph. Deep graph convolutional layers can be embedded into different tasks as a whole graph convolution module. For example, the graph convolution module followed by different node aggregation operations can be used for graph representation and graph classification tasks. it can also be used for link prediction tasks by extracting the subgraphs around the target link and feeding them into the graph classification model designed above. The graph convolution module can also extract node features for node prediction tasks, which makes the design of the model more flexible and scalable. Finally, the end-to-end graph classification model DGCNNII based on NLMP framework achieves better performance than a large number of baseline methods on 5 data sets. In the future, we hope to introduce a multi-head attention mechanism, and research frameworks and models that can better capture structural features, and expand the method to more datasets.
